# Study of Animal Mixing and the Dynamics of Hepatitis E Virus Infection on a Farrow-to-Finish Pig Farm

**DOI:** 10.3390/ani12030272

**Published:** 2022-01-22

**Authors:** Susan M. Withenshaw, Sylvia S. Grierson, Richard P. Smith

**Affiliations:** 1 Department of Epidemiological Sciences, Animal and Plant Health Agency, Weybridge KT15 3NB, UK; richard.p.smith@apha.gov.uk; 2 Department of Virology, Animal and Plant Health Agency, Weybridge KT15 3NB, UK; sylvia.grierson@apha.gov.uk

**Keywords:** pig, swine, Hepatitis E virus, HEV, zoonosis, farm, within-herd transmission

## Abstract

**Simple Summary:**

In Europe, swine are a livestock reservoir for Hepatitis E virus genotype 3 (HEV-3). Consumption of food containing HEV-3 may lead to human infection, and severe illness in some cases. Heat treatment and good hygiene practice during food preparation reduces human infection risk, but further control could be achieved by controlling HEV infection in pigs on farm. However, the key sources and timing of HEV infection in pig herds is not well understood. This study aimed to address these knowledge gaps. Pig faeces were collected from a farrow-to-finish farm on multiple occasions spanning five months and were tested for presence of HEV nucleic acid. Prevalence was always higher in growers (85.8% overall) compared to older fattener pigs (26.0%), but was detected at all visits indicating long-term persistence on the farm. Prevalence of HEV in the farm environment was also high (64.7% of 67 samples), and this may lead to continual herd re-infection. Studying infection in a single cohort of pigs over time revealed an absence of active infection in farrowing sows and young piglets. Infection first appeared in the cohort at weaner age, but only in groups that had either been weaned earlier or experienced a high degree of mixing with other pigs.

**Abstract:**

In Europe, swine are a livestock reservoir for Hepatitis E virus genotype 3 (HEV-3). Consumption of food containing HEV-3 can cause zoonotic human infection, though risk is reduced by heat treatment. Implementing controls that limit infection in slaughter pigs may further reduce foodborne transmission risk but knowledge of infection dynamics on commercial farms is limited. This study addressed this knowledge gap and in particular investigated the influence of group mixing. Faeces were collected from grower (*n* = 212) and fattener (*n* = 262) pigs on a farrow-to-finish farm on four occasions. HEV RNA was detected on all occasions, and prevalence was higher in growers (85.8%) than fatteners (26.0%; *p* < 0.001). HEV-positive samples were also collected from the wider farm environment (*n* = 67; 64.7% prevalence), indicating potential sources for HEV re-circulation within the herd. Timing of infection in a cohort was also investigated. HEV was absent from all piglet faeces (*n* = 98) and first detected at weaner stage (25.7% prevalence), but only in groups weaned earlier or comprising pigs from many different litters. Farrowing sow faeces (*n* = 75) were HEV-negative but antibodies were detected in blood from two sows. Results suggest that multiple factors influence HEV infection dynamics on pig farms, and potential foci for further study into practical control solutions are highlighted.

## 1. Introduction

Human infection with locally acquired Hepatitis E virus (HEV) is a widely recognised public health concern across Europe [1]. Strains of HEV that infect humans are classified in the species *Orthohepevirus A*, which comprises multiple genotypes (HEV-1 to HEV-8) that vary in the range of mammalian species they are known to infect [2,3]. Five genotypes (HEV-1, 2, 3, 4 and 7) are currently understood to infect humans. In Europe, human infections were once primarily associated with foreign travel to regions of Africa and Asia where genotypes HEV-1 and HEV-2 are endemic and transmission predominantly occurs via a faecal-oral route [4]. However, reports of HEV infection in people with no recent history of foreign travel have increased in number over the last decade in some European countries, and it is now well established that the virus is also endemic in this region [5]. Locally acquired human HEV infections in Europe are mainly associated with HEV genotype 3 (HEV-3) [6], which also infects pigs [7]. In fact, pigs are the main reservoir for HEV-3 worldwide [8,9,10]. Investigations across Europe have reported the presence of HEV-seropositive pigs in 30–98% of studied herds [9], and the potential for zoonotic transmission via consumption of HEV-containing pork products has been established [8,11,12,13,14].

Most human HEV infections are asymptomatic, but more serious disease can occur in immunocompromised patients and those with pre-existing liver conditions [15]. Where pork has been implicated in transmission, consumption of raw or undercooked meat or products has often been involved [8], and the risk of infection can be reduced by suitable heat treatment in domestic kitchens [16] and by other methods to inactivate the virus during food production (e.g., [17]). However, reducing the number of pigs going to slaughter that are actively infected with HEV may further reduce risk of zoonotic transmission.

Eliminating HEV infection in pigs would currently be challenging due to the apparent wide-spread nature of the virus on farms where it has been studied [18,19,20,21]. Infection does not cause overt clinical disease in pigs [7], thus infected pigs cannot be easily identified and isolated. Furthermore, the primary sources of infection and the propensity for environmental persistence are unclear, and there is no suitable HEV vaccine for pigs at present [22]. Continued within-farm circulation, resulting in reinfection of successive herds, is therefore likely on HEV-positive farms.

Maintaining a comprehensive biosecurity programme is essential to limiting HEV introduction and spread on farms. However, strategies that focus on reducing infection in slaughter-age pigs may be more attainable and economically viable in the immediate term. Existing abattoir studies demonstrate that recovery from HEV is possible, since slaughter pigs are often HEV seropositive with no obvious sign of current infection [18,19,21]. Approaches to herd management that limit opportunities for infection during later fattening stages and ensure any HEV transmission that occurs does so early in production may facilitate a high proportion of the herd clearing the infection before slaughter. The timing and extent of inter-pen mixing during the production cycle is likely to influence the rate of contact between susceptible and infected pigs and subsequent spread of infection within the pig herd, and is therefore a potential focus for such a strategy.

Prospective studies to investigate the effect of group mixing on the risk of HEV infection in pigs are currently lacking, as are farm-based investigations of within-herd HEV infection dynamics in general. Most existing studies provide snapshots of herd-level prevalence, including the few studies that have been carried out in the UK [23,24,25]. While these studies provide important evidence for the widespread occurrence of HEV, insights into risk factors for infection at different stages of production and the influence of group mixing on risk of infection at slaughter can only be gained from longitudinal investigations.

In order to address these knowledge gaps, HEV infection was investigated in a batch of farrowing sows and their offspring from piglet to slaughter age on an indoor farrow-to-finish pig farm. Within-batch differences in the degree of group mixing were recorded and the impact on HEV infection risk was assessed. Potential sources of HEV within the farm environment were also investigated and persistence within the entire herd was monitored. Together, this provides a holistic overview of HEV circulation within a commercial pig farm, and highlights key foci for further study with respect to the management of HEV on farms.

## 2. Materials and Methods

### 2.1. Study Farm

The study was carried out on a single indoor farrow-to-finish pig farm in England. The farm was selected due to its operating a closed herd (all replacement gilts bred on site and sows served via artificial insemination) and prior knowledge of the frequent between-pen mixing generally practiced, which would facilitate the study of within-farm HEV transmission. Such frequent mixing of pigs between pens is not common practice on commercial UK pig farms, making the study farm unusual in this respect (R. Davies, personal communication). In addition, overall biosecurity on the farm was minimal (e.g., absence of building-specific clothing and disinfectant boot dips, frequent pooling of water in the farmyard). As a result, levels of HEV infection identified on this study farm are not likely to reflect the wider UK situation.

Batch farrowing on the study farm took place weekly and the breeding and fattening herds were present on the same farm site. Pigs were housed across multiple pens in several different buildings, which varied in size, layout, and age. Further details of herd distribution across the farm and other aspects of the farm’s usual herd management, are given in Table S1. In general, movements of pigs between pens throughout the rearing and fattening periods were accompanied by mixing of pigs from multiple different pens, which included some re-assortment based on growth rate, though the amount of mixing at specific time points was limited to some extent by pig size and available pen capacity.

### 2.2. Study Cohort

The infection status of a selected batch of pigs, hereafter referred to as the ‘cohort’, was followed over time from piglet to slaughter age. The cohort comprised the progeny of 14 sows (the ‘cohort sows’) housed in a single farrowing house born within the same week. Throughout the rearing and fattening periods, the cohort was housed across multiple pens, and pigs were moved and mixed on several occasions. These movements and mixing were monitored using ear tags that were applied to pigs prior to weaning. Different combinations of ear tag colours and numbers were used for each litter, so that each pig’s litter of origin could be identified following mixing into weaner, grower, and fattener groups. This enabled characteristics about the composition of each cohort group, and therefore the degree of mixing that had occurred between them, to be determined at each stage of production, including the number of cohort litters represented in each group. In addition, pigs from different farrowing houses that were not part of the original cohort (and so were not ear-tagged) could be identified if mixed with cohort pigs at weaning. The proportion of untagged (‘non-cohort’) pigs per group could therefore be calculated at each production stage, to provide a measure of mixing between cohort and non-cohort pigs.

A detailed description of the movement and mixing of groups of cohort pigs over time is illustrated in Figure S1. Cohort litter size varied between 9 and 12 pigs per sow, and the cohort initially comprised 153 pigs. At weaning (age approximately 4 weeks), pigs from different cohort litters were mixed together and with 100 untagged non-cohort pigs (born in a different farrowing house) in an ad hoc manner and split into seven pens in a weaner building where they were held for the next seven weeks. Each group (hereafter referred to as wn1–wn7) consisted of between 32 and 34 pigs per pen, and groups varied in terms of the number of different cohort litters represented (1–9 different litters per group) and the ratio of cohort to non-cohort pigs (0–72.7% per group being non-cohort pigs). At the end of this period, most non-cohort pigs that had joined the study were again separated from the cohort and moved elsewhere on the farm. The remaining pigs were moved and mixed into two groups of growers: group gr1 comprised 101 pigs housed in a single large pen bedded on deep straw, and group gr2 comprised 43 pigs housed in a slatted-floor pen in a different building. In contrast to the farm’s usual practice of moving pigs to smaller pens for the latter stages of fattening (see Table S1), group gr1 underwent no further movement or mixing prior to slaughter at age 21–22 weeks, though reduced in size to 94 pigs by this time due to mortality. In contrast, pigs in group gr2 were divided across multiple fattening pens at approximately 19 weeks of age, though were subsequently lost from the study because of earlier than expected slaughter.

### 2.3. On-Farm Sample Collection

Individual floor faecal samples were collected from pig pens and tested for the presence of HEV RNA. Details of the sampling scheme, including the timing of sampling visits and the groups of pigs sampled at each visit are given in Table 1. Five sampling visits were carried out between May and October 2018. The timing of the visits coincided with key events during the rearing of the cohort (see Figure S1). Samples were collected from cohort sows approximately one week prior to farrowing (visit 1 in early May; three replicate faecal samples per sow) and then again just prior to piglet weaning (visit 2 in late May; two replicate faecal samples per sow). Faecal samples were collected from all pens containing the cohort as piglets aged 3–4 weeks (visit 2), as weaners aged 11–12 weeks (visit 3 in July), as growers aged 18–19 weeks (visit 4 in August), and finally as fatteners approximately one week prior to slaughter when aged 22–23 weeks (visit 5 in October). In addition to samples collected from the cohort, faecal samples were also collected from all other occupied pens of growers and fatteners present on the farm (hereafter referred to as ‘non-cohort’ growers and fatteners) during visits 1, 3, 4 and 5 (Table 1). All faecal samples comprised approximately 50 g of faeces collected from a discrete faecal pat. Further methodological details of faecal sample collection are described in Appendix A.

Samples from the farm environment were also collected at each visit and tested for the presence of HEV RNA. Sample types varied between visits, reflecting substrate availability, but typically included pooled farmyard water, wildlife faeces (rodents and wild birds), and swabs of drinkers/feeders, dust, pen walls, pig herding boards, forks/spades for mucking out pig pens, farm workers’ personal protective clothing, and tractor tyres and foot wells. Further methodological details of environmental sample collection and pre-storage laboratory processing are described in Appendix B.

### 2.4. Post-Mortem Sampling of Cohort Sows

Post-mortem sampling was carried out on two available cohort sows in August and October 2019. Due to the study farm being a commercial enterprise, post-mortem sampling could only take place once each sow had reached their usual culling age. Only two of the cohort sows reached this age within the time frame of the study, which was approximately 15 months and 17 months since the last faecal samples were collected from these animals on-farm, respectively. Each sow was seventh parity by this time, and approximately 54 months old. The sows were transported to the Animal and Plant Health Agency, where they were culled in accordance with Schedule 1 of the UK Animals (Scientific Procedures) Act 1986 [26]. A blood and bile sample was collected from each sow, along with the whole liver which was separated into left and right lateral and medial lobes. Isolated serum, bile, and tissue samples were stored at −80 °C until further processing. Resource constraints meant that blood sampling of live pigs was not possible during this study, therefore the purpose of this post-mortem sampling was to obtain additional evidence relating to previous and current HEV infection in the farm’s breeding herd which would not be evident from the results of floor faecal sampling alone.

### 2.5. Laboratory Detection of HEV RNA and Antibodies

Nucleic acid was extracted from faeces and non-swab environmental samples collected on farm using the LSI MagVet™ Universal Isolation Kit (Life Technologies Ltd., Paisley, UK) and from post-mortem samples of blood, bile, and liver using the Viral RNA Mini kit (QIAGEN, Manchester, UK) according to manufacturer’s instructions. For environmental swab samples, nucleic acid was extracted from clarified (10,000 g for 2 min at 4 °C), concentrated (10×) swab elute using the MagMAX™ Pathogen RNA/DNA Kit (Life Technologies Ltd., Paisley, UK) according to the manufacturer’s instructions. Detection of host RNA (b-actin) [27] was used to demonstrate extraction of amplifiable nucleic acid in samples of faeces, blood, bile, liver, and certain types of environmental samples (faeces and dust/sludge). For all other sample types, Internal Control RNA (QIAGEN, Manchester, UK) was added during nucleic acid extraction to enable the extraction of amplifiable RNA to be demonstrated.

Extracts were tested for the presence of HEV RNA using a TaqMan real-time RT-PCR, which detects genotypes 1–4 of *Orthohepevirus A* [28]. For faeces, HEV copy number per gram (cn/g) was estimated using titration of the WHO International Standard (Paul-Ehrlich-Institut (PEI) code 6329/10, Paul-Ehrlich-Institut, Germany) [29]. In brief, log10 serial dilutions of the standard nucleic acid that had been extracted using the LSI MagVet™ Universal Isolation Kit (Life Technologies Ltd., Paisley, UK) were tested in parallel with samples. Copy number in samples was estimated by extrapolation from the standard curve and with a multiplication factor (×5) to account for sample dilution in the extraction protocol (manufacturer’s instructions). Absolute quantitation of HEV RNA was not applied for environmental samples because the precise nature of the swab material collected could not be determined given the methods used, and so detected/ not-detected only is reported. Serum samples collected from the two cohort sows at post-mortem were tested for the presence of total HEV antibodies using the HEV Ab ELISA (Fortress Diagnostics, Antrim, UK) according to the manufacturer’s instructions.

### 2.6. Data Analyses

#### 2.6.1. Farm-Level Trends in Faecal HEV Presence and Viral Load

Broad farm-level trends in the presence of HEV were investigated by comparing HEV presence in the faeces of non-cohort growers and fatteners over time using a binomial generalised linear model (GLM) with a logit link. Pig stage (a factor with two levels: grower and fattener) and visit month (a factor with four levels: May, July, August, October) were investigated as explanatory variables. An interaction between pig stage and visit month was included to allow for the possibility of differences in temporal trend between pig stages. A backwards stepwise procedure was then used to identify significant terms, with the interaction term being the first term dropped if not significant.

The same variables were investigated in a two-way Analysis of Variance (ANOVA) to investigate differences in the viral load of HEV-positive faeces collected from non-cohort growers and fatteners over time. Viral load was highly right-skewed; therefore log_10_ of HEV cn/g was used as the normalised dependent variable. A post hoc Tukey test was used to determine which groups differed significantly in their mean viral load. All analyses were carried out in the base package of R v3.4.4 [30], and the threshold for significance was set at 5% for all statistical tests.

#### 2.6.2. Dynamics of HEV Faecal Shedding in the Cohort over Time

A similar modelling framework was used to investigate whether faecal HEV presence changed over time as pigs grew older and progressed through production. Using data from cohort pigs only, age was investigated as the single fixed effect in a GLM. A one-way ANOVA was used to investigate if the viral load of positive faecal samples differed between cohort age groups. The dependent variable was log_10_ of HEV cn/g, and a post hoc Tukey test was used to determine which age groups differed significantly.

#### 2.6.3. Effect of Inter-Group Mixing on Faecal HEV Presence and Viral Load in Cohort Weaners

The effect of prior mixing on the risk of HEV faecal shedding was investigated by comparing the presence of HEV in faeces across different pens of cohort pigs at the same sampling occasion. Due to a lack of variation in HEV presence across cohort pens at grower and fattener stages (see Results), this comparison was only possible for cohort weaners after they had experienced a single mixing event. Presence of HEV in the faeces of cohort pigs sampled as weaners during visit 3 was modelled using a binomial Generalised Linear Mixed Model (GLMM) using the lme4 package in R [31], with pen ID included as a random effect to account for the non-independence of samples collected from the same pen. Three fixed effects were investigated in separate univariable models, each of which reflected a different measure of the degree of mixing previously experienced by pigs within each pen: (1) Number of different cohort litters per group as a continuous variable, (2) Number of different cohort litters per group as a categorical variable with two levels (≤5, >5), and (3) Proportion of non-cohort pigs per group. Welch’s *t*-test was also used to determine if HEV viral load in faeces differed significantly between pens.

## 3. Results

### 3.1. Farm-Level Trends in Faecal HEV Presence and Viral Load

**Figure 1:**
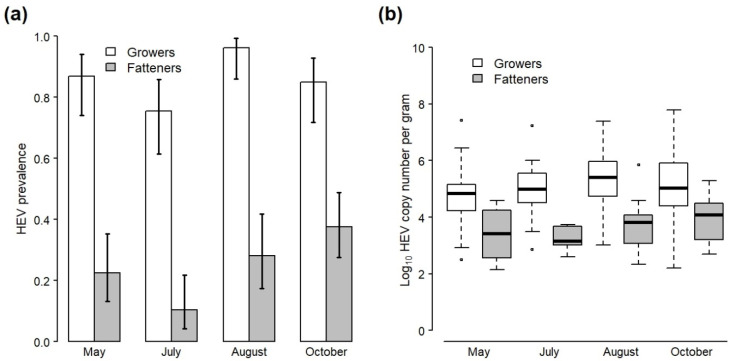
(**a**) Prevalence and 95% confidence intervals of HEV RNA in all faecal samples collected from the pens of grower and fattener pigs during four visits to the study farm. (**b**) Boxplots of viral load (log_10_ HEV copy number per gram) in HEV-positive faecal samples taken from the pens of grower and fattener pigs during four visits to the study farm (stars represent outliners). Data do not include samples collected from the study cohort, which are presented separately in Figure 2.

Across all sampling visits, 474 faecal samples were collected from non-cohort growers and fatteners. In total, 85.8% of samples from growers (182/212) tested positive for HEV RNA compared with 26.0% from fatteners (68/262) (Table 2). The genomes of a selection of HEV RNA-positive samples collected throughout the study were partially characterized using sanger sequencing (*n* = 36) and all were confirmed as HEV-3 (data not shown). Significant effects of pig stage (χ^2^ = 193.0, df = 1, *p* < 0.001) and sampling month (χ^2^ = 19.44, df = 3, *p* < 0.001) were identified with respect to HEV detection according to the GLM, but there was no significant interaction between them (χ^2^ = 5.93, df = 3, *p* = 0.12). HEV was significantly more likely to be detected in growers compared to fatteners across all sampling visits (Odds Ratio (OR) 21.6; 95% confidence interval (CI) 13.1—37.1) and less likely to be detected in July (Visit 3) compared to other months for both pig stages (OR 0.45; CI 0.22–0.89) (Figure 1a; Table S2).

Mean viral load in positive faecal samples across all visits was 5.05 log_10_ cn/g for growers and 3.73 log_10_ cn/g for fatteners (Table 2; Figure 1b). A two-way ANOVA found no significant interaction between the effect of pig stage and sampling month on viral load (*F*_3,242_ = 1.12, *p* = 0.34), but there were separate significant effects of pig stage (*F*_1,242_ = 97.8, *p* < 0.001) and sampling month (*F*_3,242_ = 3.72, *p* = 0.01) (Table S3). A post hoc Tukey test found that viral load was significantly higher in growers compared to fatteners across all sampling visits (*p* < 0.001) and significantly higher in August (visit 4) compared with early May (visit 1) across both pig stages (*p* = 0.01), but pairwise differences between other sampling months were not significant (Table S4).

### 3.2. Presence of HEV in Sows

A total of 45 faecal samples were collected from sows during visit 1 in May, and 30 more samples were collected during visit 2 four weeks later. None of these samples had detectable HEV RNA. Samples were collected from 18 sows in total, including the 14 cohort sows plus four sows involved in piglet cross-fostering. Twelve of the sows were sampled at both visits.

Samples of serum, bile, and liver were collected from two cohort sows during post-mortem examination. Both of these cohort sows had detectable HEV antibodies in serum. Neither sow had detectable HEV RNA in any sample types (serum, bile, or liver).

### 3.3. Dynamics of HEV Faecal Shedding in the Cohort over Time

**Figure 2:**
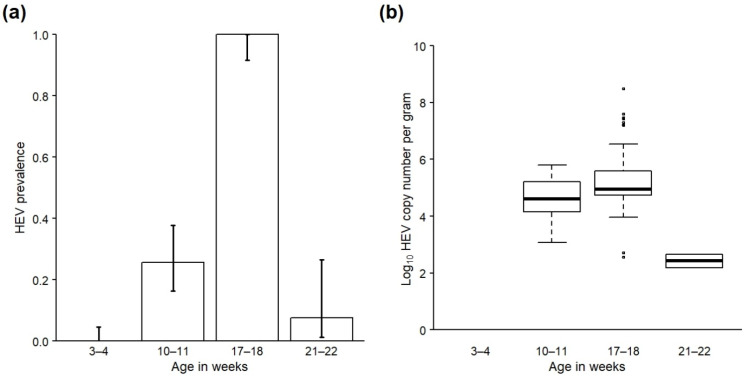
(**a**) Prevalence and 95% confidence intervals of HEV RNA in all faecal samples and (**b**) viral load (log_10_ copy number per gram) in HEV-positive faecal samples taken from a cohort of pigs on a farrow-to-finish farm in England when sampled at 3–4 weeks, 10–11 weeks, 17–18 weeks and 21–22 weeks of age.

HEV was not detected in any faecal samples collected from the cohort as piglets (0/98). However, prevalence was 25.7% (18/70) in samples collected from the cohort eight weeks later as weaners (Table 3; Figure 2a), and log_10_ of the mean viral load (cn/g) of positive samples was 4.70 (range 3.07–5.80) (Table 3; Figure 2b). Prevalence increased again by the time the cohort was sampled seven weeks later as growers, with 100% of samples (53/53) now testing positive, and log_10_ of the mean viral load also increased to 5.31 (range 2.56–8.49). There followed a large decrease in prevalence and viral load by the time of sampling shortly prior to slaughter; only 7.7% of samples (2/26) tested positive at this time, and log_10_ of the mean viral load in these positive samples was 2.42 (range 2.19–2.65). A detailed description of how groups of cohort pigs were moved and mixed on the farm during production is given in Appendix C and is graphically presented in Figure S1. Notably, most cohort pigs were housed in a single large (approximately 100 pigs) stable group for the 10 weeks prior to slaughter, which contrasts with the farm’s usual practice (compare with Table S1 which gives detail of the farm’s usual herd management practice).

There was a significant effect of age on the presence of HEV in cohort faeces according to the GLM (χ^2^ = 24.8, df = 1, *p* < 0.001), with the likelihood of HEV presence being significantly lower by slaughter age (22–23 weeks old) compared with younger age categories (OR: 0.06; CI: 0.01–0.22; Table S5). Age was initially modelled as a factor with three levels (11–12 weeks, 17–18 weeks and 21–22 weeks), but this model resulted in over-inflated standard errors due to 100% of samples from one of the age categories being positive. The final model therefore included cohort age as a factor with two levels (11–12 and 17–18 weeks (combined) and 21–22 weeks). Since HEV was not detected in the cohort when sampled as 3–4-week-old piglets, the results of these samples were not included in the model as it resulted in perfect prediction.

There was also a significant effect of age on viral load in HEV-positive cohort faeces according to the one-way ANOVA (*F*_2,70_ = 8.50, *p* < 0.001; Table S6). A post hoc Tukey test identified that mean viral load was significantly lower by slaughter age compared to when sampled as 17–18-week-old growers (*p* = 0.001) and 11–12-week-old weaners (*p* = 0.02), but the difference in viral load between weaner (11–12 weeks) and grower (17–18 weeks) time points was not significant (*p* = 0.10; Table S7).

### 3.4. Effect of Inter-Group Mixing on Faecal HEV Presence and Viral Load in Cohort Weaners

By the age of 10–11 weeks the cohort was split into seven groups housed in separate pens (see Appendix C and Figure S1). These groups are hereafter referred to as wn1–wn7 and details of group size and composition are presented in Table 4. Groups were similar in size (median 33; range 32–34 pigs per pen) but pigs from different cohort litters were mixed together in an ad hoc manner to form each group (Figure S1). The number of cohort litters represented within each group therefore varied and ranged from one to nine (median 3). Furthermore, 30.3–72.7% of pigs in groups wn2–wn7 were not part of the original study cohort, having been mixed with the cohort at the point of weaning, though there were no such ‘non-cohort’ pigs in group wn1.

HEV was detected in 2/7 cohort weaner groups (groups wn1 and wn2), with 90.0% of faecal samples (9/10) testing positive in each of these groups. Log_10_ mean viral load of positive samples was 4.47 (range 3.07–5.80) for group wn1 and 4.93 (range 4.09–5.75) for group wn2, and the difference between the two was not significant according to a two-tailed *t*-test (*t* = −1.34, d.f. = 15.1, *p* = 0.20). None of the measures of prior mixing that were investigated (number of different cohort litters per weaner group and proportion of non-cohort pigs per group) were significant predictors of HEV presence in the faeces of cohort weaners overall (Table S8). However, the composition of each HEV-positive group was notably different compared with the HEV-negative groups. Group wn2 was composed of the greatest number of different cohort litters (*n* = 9) and also included a high percentage of non-cohort pigs (56.3%) (Table 4). Group wn1 was composed of relatively few different cohort litters (*n* = 3) and no non-cohort pigs. However, the pigs in this group had been weaned and mixed together into this larger group at least five days earlier than the cohort pigs in other groups (see Appendix C and Figure S1 for details) and the same group tested negative for HEV when sampled seven weeks earlier shortly after weaning.

### 3.5. Presence of HEV in the Farm Environment

Across all sampling visits, 76 samples were collected from the farm environment. Due to the presence of inhibitory compounds, nine samples could not be tested for the presence of HEV RNA. In total, 64.2% of samples tested positive (43/67; Table 5). The positive samples included a variety of substrates including: swabs of pig herding boards (3/3 samples positive), a swab of an indoor walkway, swabs of the tyres and foot wells of farm vehicles (9/10 samples positive), farmyard surface water (13/15 samples positive), surface dust from inside occupied pig housing (3/4 samples positive), hand-held farm tools such as shovels, forks and ear taggers (5/8 samples positive), drinkers, feeders and enrichment toys inside occupied pig pens (4/7 samples positive), and composite rat faeces (2/5 samples positive) (Table 5). In addition, all three surface swabs taken from a recently cleaned and disinfected pen tested positive. In contrast, three swab samples were collected from an outdoor tap on three separate occasions and none of these samples tested positive. A swab of a hand-held ear tagging device located in the farrowing house also tested negative, as did all samples of mouse faeces (*n* = 2), wild bird faeces (*n* = 4) and domestic dog faeces (*n* = 1).

Positive environmental samples were collected from the vicinity of all production stages (Table 5). Samples collected from in and around the grower buildings had the highest rate of HEV positivity (71.4%), but more than half of the samples collected in the areas around the fattener and weaner buildings also tested positive (55.6% and 53.8%, respectively). Overall, 40.0% of environmental samples from the farrowing houses tested positive. However, it was notable that none of the surface water samples collected from near to the farrowing houses tested positive, despite 100% of such samples testing positive from areas nearby the grower and fattener buildings (Table 5).

## 4. Discussion

This study provides a holistic overview of HEV circulation on pig farms. Despite HEV presence on the study farm being unknown prior to the investigation, viral RNA was detected in faecal and environmental samples collected from the farm on all sampling occasions. This result adds to the existing body of evidence indicating that HEV is widespread in pigs in countries where it has been studied [9].

Sampling of the cohort over time revealed age-associated variation in the shedding of HEV in faeces, which provides indirect evidence of the dynamics of infection within the pig herd on this farm. HEV RNA was first detected in cohort faeces when sampled as 11–12-week-old weaners, having been absent from faeces when first sampled at 3–4 weeks of age. Since data from the two sows that were analysed demonstrated the presence of anti-HEV antibodies in sera, it is likely that maternal antibodies play a role in the delayed onset of infections.

However, the length of time between HEV infection and the onset of faecal shedding for naturally infected pigs is currently uncertain, though has been estimated at between 1–2 weeks in experimental infection studies [32,33,34,35]. It is, therefore, also possible that some cohort pigs were infected but not shedding when sampled at 3–4 weeks of age. Indeed, viral shedding in faeces has been detected previously in piglets as young as 3 weeks old on a farrow-to-finish farm in Spain [36]. Interestingly, however, when HEV was eventually detected in the present study cohort for the first time, it was present in only two of seven groups of weaners, suggesting that different groups of pigs became infected at different times.

Understanding why some pigs become infected earlier than others is important for managing the dynamics of HEV infection within pig herds and reducing active infection at slaughter. A lack of statistical power meant that significant drivers of variation in HEV shedding between cohort weaner groups could not be identified here. However, it was notable that pigs in one of the earlier shedding groups had experienced a greater degree of group mixing during early life compared with other groups (though the presence of non-cohort pigs from an unknown number of litters meant that the absolute variation between groups in the amount of prior mixing was unmeasurable). In silico studies have demonstrated that within-herd contact structure influences the rate of HEV transmission in pigs [37], with between-pen mixing being associated with higher rates of transmission. A retrospective risk factor study involving data from 90 pig farms in France also found that mixing groups of piglets together at an intermediate stage between farrowing and nursery was associated with higher farm-level seroprevalence at slaughter, thus indicating more widespread infection when pigs were mixed earlier in life [38]. Most cohort pigs in the present study were subsequently mixed into a single large group for the remaining rearing and fattening period. Therefore, although not possible to monitor how differences in the degree of mixing earlier in life directly influenced infection status at slaughter, these preliminary results clearly indicate the value of further study in this area.

Pigs in one other cohort group were found to be shedding HEV in their faeces at 11–12 weeks of age. This group had experienced arguably the least amount of pre-nursey mixing, being composed of pigs from just three litters and having not been mixed with any pigs from outside of the original study cohort. Earlier exposure to infection is therefore unlikely to explain why pigs in this group were apparently infected earlier than most others. However, all of these pigs were weaned earlier than most others; they may therefore have been more susceptible to infection from an earlier time point following an initial period of immunity afforded by the presence of maternal antibodies. HEV-specific maternal antibodies can be passed from seropositive sows to offspring via colostrum [7,36,39]. The antibody status of weaners was not determined in this study; however, two maternal cohort sows were positive for total HEV antibodies when blood was tested post-mortem. This result should be interpreted with some caution due to the very small sample size; however, given that all replacement gilts were reared on the farm and the consistent presence of HEV in the herd and farm environment (including in the vicinity of farrowing houses), it is likely that the majority of farrowing sows were seropositive. The presence of maternal antibodies can influence the dynamics of infection in offspring [40] and may even delay the onset of viraemia [41]; this may be why shedding was absent in the cohort when sampled at 3–4 weeks of age. However, physiological effects of increased stress resulting from earlier weaning may diminish the immune response [42] and could have led to increased susceptibility and earlier infection in this group of pigs once the maternal antibody protection waned. However, measurement of antibody concentrations in sows and offspring would be required to assess this directly.

Following initial infection, the virus appeared to rapidly spread throughout the cohort with an estimated prevalence of 100% by the time the pigs were sampled at 17–18 weeks old. This mirrors the similarly high prevalence of infection consistently seen across all growers on the farm. Rapid dissemination of infection through the herd was also demonstrated in a farm study that followed HEV infection dynamics on a farrow-to-finish farm in Spain [39], with most sampled pigs shedding HEV in their faeces within three weeks of the first pig shedding the virus. In the current study, rapid dissemination of the virus within the study cohort may have been facilitated by the movement and mixing of pigs into larger groups following the nursery period. Most pigs in the study cohort were mixed into a single very large group, so enabling frequent contact between infected and susceptible pigs and facilitating virus spread.

In addition to frequent pig-to-pig contact, most cohort pigs were bedded on deep straw from approximately 12 weeks of age. This may have provided ideal conditions for the survival and accumulation of HEV within the pen environment, so further facilitating indirect within-pen transmission. Indeed, in an experimental study, Andraud et al. [35] demonstrated that the quantity of HEV present in the pen environment as a result of faecal shedding by infected individuals strongly influenced the rate of within-pen transmission. Furthermore, Walachowski et al. [38] found that within-herd seroprevalence was significantly higher on pig farms in France where the gap between pit manure and the slatted floor of fattening rooms was small, which presumably resulted in more frequent contact between pigs and contaminated faeces. This may also help to explain why the virus was consistently widespread across the entire rearing herd on this farm, even though most pigs were housed in smaller groups throughout the rearing period. Except for one building which had slatted floors, the rearing herd was straw-bedded on solid floors with a sweep-through system in operation for removing waste from dunging channels. This, along with the continuous-flow management and relatively infrequent cleaning and disinfection of grower and fattener pens on this farm (see Table S1), may have facilitated the spread of contaminated waste between pens and persistence of HEV on the farm.

The peak in HEV prevalence in the cohort during the mid-stages of rearing at 17–18 weeks of age was followed by a rapid decline by the age of 21–22 weeks. A handful of other farm studies have investigated natural HEV infection dynamics longitudinally and some have reported similar herd-level infection dynamics to those presented here. In Canada, Leblanc et al. [43] followed 51 pigs from a single cohort in a simulated commercial farm setting and found that prevalence of faecal HEV shedding increased over time to a peak of 86% by the age of 18 weeks, and subsequently declined to 41% by slaughter. Similarly, de Deus et al. [39] followed pigs from a single batch on a farrow-to-finish farm in Spain and demonstrated a peak in the prevalence of HEV RNA in serum samples at 15 weeks of age (42% of 26 pigs), followed by a reduction by the final sampling point at 22 weeks of age (13% of 16 pigs). Although several experimental studies have demonstrated an indirect relationship between HEV viraemia and rate of faecal shedding (e.g., [32,34,35,44]), the fact that this decline in faecal shedding followed a striking peak in prevalence is consistent with the notion of initial widespread infection in the cohort followed by recovery.

In addition to following the cohort through time, broader insights into HEV persistence and circulation within pig farm environments can be identified from this study. Cross-sectional sampling of all other grower and fattener pigs on four different occasions demonstrated that HEV was clearly established within the study farm, with a consistently high prevalence (at least 75% per sampling occasion in growers) sustained across the five-month study period. Husbandry practices including frequent group mixing as well as low levels of biosecurity have previously been identified as favouring within-herd persistence of HEV in transmission models [35]. Frequent mixing and movement of pigs between buildings coupled with relatively low biosecurity practices therefore likely facilitate the continued circulation of the virus in successive herds on this farm, leading to the persistence of infection seen within the herd overall.

Prevalence of HEV in the faeces of all grower and fattener pigs was significantly lower in July compared with other months, which could suggest seasonal differences in the occurrence of HEV infection in the herd. This contrasts with the results of an existing retrospective study of slaughter pigs in France, which found that season (summer, autumn, winter, spring) did not affect HEV seroprevalence at slaughter or HEV prevalence in livers at slaughter at individual or farm level [19]. However, the relatively short duration of the current study means that robust conclusions about seasonality cannot be drawn, and it is possible that the temporal differences identified merely reflect differences in the overall age structure of the herd when sampled at different time points rather than an extrinsic effect of season. Interestingly, however, a study of HEV seroprevalence in Mexico found that seropositivity was significantly associated with mean annual rainfall, with higher odds of pig seropositivity in areas that experienced <600 mm of rainfall per year [45]. Seasonal and climatic factors therefore do have the potential to influence the dynamics of HEV infection, and further studies of within-farm prevalence carried out over an extended time are needed to ascertain the relative importance of such effects in temperate regions of Europe.

A high proportion of environmental samples collected from across all areas of the farm tested positive for HEV RNA in this study, indicating widespread environmental contamination which may exacerbate continued circulation in the herd. For example, several previous studies have found that persistence of HEV within farm wastes is possible [24,46,47]. Given that most pooled water samples collected here tested positive for HEV RNA, dissemination of the virus via waste run-off seems highly probable. HEV RNA was also detected in rat faeces, and while rodents have previously been identified as passive carriers of HEV infection rather than being actively infected themselves [48], this nevertheless represents another means of viral dissemination within this farm. Together, these results reinforce the fact that successful management of HEV on pig farms requires robust biosecurity practices to be maintained in addition to any potential herd management controls.

The viability of HEV in environmental samples collected in this study was not determined. However, Kasorndorkbua et al. [47] found evidence of viable HEV in slurry samples from a pig farm in the mid-west USA, as inoculation of pathogen-free pigs resulted in seroconversion and subsequent faecal shedding in these animals. Nevertheless, further studies to demonstrate the viability of HEV within environmental samples collected on European farms are needed but are currently limited by a lack of reliable in vitro infectivity assay [49], which necessitates the use of costly, time-consuming bioassay experiments, which are rarely a feasible study option.

## 5. Conclusions

This study provides a holistic overview of HEV circulation on a farrow-to-finish pig farm. It provides insights into herd-level infection dynamics, within-herd persistence over time, and the potential role of environmental contamination in continued on-farm circulation which is broadly applicable to controlling HEV on pig farms across pork-producing nations. Identifying relevant, practical farm management solutions to controlling the on-farm circulation of HEV may contribute to minimizing the risk of foodborne human HEV infection alongside existing control measures. Further on-farm investigations to determine how biosecurity practices affect HEV presence and persistence, and how herd infection dynamics vary across different types of farm, including integrated breeding and finishing farms that mix groups of pigs together at different stages of production, are required to confirm and extend our study.

## Figures and Tables

**Table 1:** Details of the study sampling scheme, including sampling dates and the groups of pigs sampled during each visit.

Cohort or Non-Cohort	Pig Groups Sampled	Visit 1 1 May 2018	Visit 2 29 May 2018	Visit 3 16 July 2018	Visit 4 31 August 2018	Visit 5 2 October 2018
Cohort	Sows	✓	✓			
	Piglets		✓			
	Weaners			✓		
	Growers				✓	
	Fatteners					✓
Non-cohort	Growers	✓		✓	✓	✓
	Fatteners	✓		✓	✓	✓
Farm environment	n.a.	✓	✓	✓	✓	✓

**Table 2:** HEV prevalence and viral load in floor faecal samples collected from non-cohort grower and fattener pigs on four occasions on a farrow-to-finish pig farm in England. Sampling was cross sectional, and different pigs were sampled on each occasion.

Production Stage	Visit No. (Month)	HEV Prevalence (%) [C.I.] ^1^ (No. Positive Samples/Total)	HEV Viral Load ^2^ (log_10_(Copy Number/g))		
			Range	Mean	Variance
Non-cohort growers	1 (May)	86.8 (46/53) [74.0–94.1]	2.50–7.43	4.78	0.80
	3 (July)	75.4 (40/53) [61.4–85.8]	2.87–7.24	4.98	0.60
	4 (August)	96.2 (51/53) [85.9–99.3]	3.01–7.39	5.37	0.99
	5 (October)	84.9 (45/53) [71.9–92.8]	2.20–7.78	5.04	1.53
	All visits	85.8 (182/212) [80.3–90.1]	2.20–7.78	5.05	1.02
Non-cohort fatteners	1 (May)	22.6 (14/62) [13.3–35.3]	2.16–4.60	3.42	0.77
	3 (July)	10.3 (6/58) [4.28–21.8]	2.61–3.73	3.23	0.18
	4 (August)	28.1 (16/57) [17.4–41.4]	2.33–5.86	3.74	0.71
	5 (October)	37.6 (32/85) [27.6–48.9]	2.69–5.28	3.95	0.57
	All visits	26.0 (68/262) [20.8–31.8]	2.16–5.86	3.73	0.65

^1^ 95% confidence intervals; ^2^ HEV-positive faecal samples only.

**Table 3:** HEV prevalence and viral load in floor faecal samples collected from a cohort pigs on four occasions during their production on a farrow-to-finish pig farm in England.

Cohort Age	Visit No. (Month)	HEV Prevalence (%) [C. I.] ^1^ (No. Positive Samples/Total)	HEV Viral Load ^2^ (log_10_(Copy Number/g))		
			Range	Mean	Variance
3–4 weeks	2 (May)	00.0 (0/98) [00.0–4.70]	na	na	na
10–11 weeks	3 (July)	25.7 (18/70) [16.3–37.8]	3.07–5.80	4.70	0.55
17–18 weeks	4 (August)	100 (53/53) [91.6–100]	2.56–8.49	5.31	1.37
21–22 weeks	5 (October)	7.7 (2/26) [1.34–26.6]	2.19–2.65	2.42	0.11

^1^ 95% confidence intervals; ^2^ HEV-positive faecal samples only.

**Table 4:** The composition of each of seven groups of cohort pigs sampled as weaners (10–11 weeks). Ten floor faecal samples were collected per group and tested for HEV RNA. Positive samples were detected in just two groups; these groups are marked with ‘(+)’.

Group ID	Group Size ^1^	No. Cohort Litters ^2^	No. Non-Cohort Pigs (%) ^3^
wn1(+)	32	3	0 (00.0)
wn2(+)	32	9	18 (56.3)
wn3	33	6	17 (51.5)
wn4	33	4	10 (30.3)
wn5	34	2	17 (50.0)
wn6	33	1	24 (72.7)
wn7	33	2	14 (42.4)

^1^ Number of pigs in the group. ^2^ Number of different cohort litters that were mixed at weaning to form the group. ^3^ Number and percentage of pigs in the group that were not part of the original study cohort.

**Table 5:** The number of environmental samples collected across five sampling visits to a farrow-to-finish pig farm in England that tested positive for HEV, categorised by sample type and general location on the farm. Sample types are ordered from highest to lowest % of positive samples.

Sample Type	No. Positive Samples/No. Collected from Each General Farm Location ^1^					Total (%)
	FS	W	G	F	Vehicles	
Surfaces inside cleaned pens			3/3			3/3 (100)
Pig herding boards		1/1	1/1	1/1		3/3 (100)
Indoor walkways		1/1				1/1 (100)
Tyres, foot wells					9/10	9/10 (90.0)
Farmyard surface water	0/2		9/9	4/4		13/15 (86.7)
Indoor surface dust	1/1			2/3		3/4 (75.0)
Hand-held farm tools	1/1	1/2	2/2	1/3		5/8 (62.5)
Drinkers/feeders/toys		3/3		1/4		4/7 (57.1)
Composite rat faeces		1/2	0/2	1/1		2/5 (40.0)
Composite mouse faeces				0/2		0/2 (00.0)
Composite wild bird faeces		0/4				0/4 (00.0)
Single domestic dog faeces			0/1			0/1 (00.0)
Ear tagger	0/1					0/1 (00.0)
Outdoor tap			0/3			0/3 (00.0)
All sample types	2/5 (40.0%)	7/13 (53.8%)	15/21 (71.4%)	10/18 (55.6%)	9/10 (90.0%)	43/67 (64.2%)

^1^ Categorized according to which pig production unit was closest to the sampling location; FS: farrowing sows; W: weaners; G: growers; F: fatteners.

## Data Availability

The data presented in this study are available on request from the corresponding author. The data are not publicly available due to privacy restrictions.

## Supplementary Materials

The following are available online at https://www.mdpi.com/article/10.3390/ani12030272/s1, Table S1: Details of the usual pig herd management on the study farm, Table S2: Results of a GLM investigating the effect of pig stage and sampling month on the presence of HEV RNA in pig faeces, Table S3: Results of a two-way ANOVA investigating the effects of pig stage and sampling month on the viral load of HEV in the faeces of grower and fattener pigs, Table S4: Results of a post hoc Tukey test to identify the sampling months that differed significantly in terms of the mean viral load of HEV in the faeces of grower and fattener pigs, Table S5: Results of a GLM investigating the effect of age on the presence of HEV RNA in faeces collected from a cohort of pigs from piglet to slaughter age, Table S6: Results of a one-way ANOVA investigating the effect of pig age on the viral load of HEV in the faeces of a cohort of pigs from piglet to slaughter age, Table S7: Results of a post hoc Tukey test to identify the age points that differed significantly in terms of the mean viral load of HEV in the faeces of a cohort of pigs, Table S8: Results of three different univariable mixed-effects logistic regression models investigating risk factors associated with the occurrence of HEV in faecal samples collected from seven groups of 17–18 week old weaner pigs, Figure S1: A diagrammatic representation of the mixing of cohort pigs throughout the study and the corresponding timing of sampling visits.

## Appendix A

Further methodological details of faecal sample collection: All faecal samples were collected into sterile universal tubes using a sterile spoon. The number of samples collected per pig stage at each visit was determined by reference to previously published estimates of HEV prevalence in the UK [24] and ensured that HEV prevalence in the cohort, and in non-cohort growers and fatteners, could be determined with at least 15% precision and 95% confidence. A systematic approach to sampling was used, with at least one sample being collected per pen, and the number of samples per pen being proportional to the number of pigs per pen. In addition, the number of samples collected per visit from each cohort pen ensured the detection of at least one positive sample if present at a prevalence of at least 25% in weaners, 45% in growers and 10% in fatteners. Once collected, samples were returned to the laboratory and stored at −80 °C until further processing.

## Appendix B

Further methodological details of environmental sample collection and pre-storage laboratory processing: Samples of pooled water, wildlife faeces and indoor surface dust/sludge were collected directly into sterile plastic sampling pots. Other types of environmental sample were collected using sterile cotton swabs (100 cm^2^ surface area) soaked in 20 mL of PBS containing Gentamicin, which were then placed into a sterile container. Samples were returned to the laboratory and non-swab samples were stored at −80 °C until further processing. Swab samples were stored at 4 °C and processed within 72 h of receipt, whereby swab contents were manually eluted into the PBS and the clarified supernatant (3000 g for 5 min at 4 °C) was stored at −80 °C until further processing.

## Appendix C

Detailed description of the movement and mixing of groups of cohort pigs over time: Cohort litter size varied between 9–12 pigs per sow, and in total the cohort comprised 153 pigs when sampled for the first time as piglets (visit 2, late May). The majority of litters (10/14) were un-weaned at this time, but four litters had been weaned and moved from their farrowing pens. One litter, weaned two days prior to sampling, was located in a different farrowing house but had not been mixed with other pigs. Three further litters, weaned seven days prior to sampling, were located in a pen in the weaner building and had been mixed together into a single group (group wn1). Shortly after this sampling visit, all remaining cohort pigs were moved into the weaner building.

The cohort was sampled again after being housed in the weaner building for seven weeks (or eight weeks in the case of group wn1) (visit 3, July). Since the previous visit, 23 of the original cohort pigs had either died or been moved elsewhere on the farm and were not included further in the study. In addition, the remainder of the cohort had been mixed with 100 untagged ‘non-cohort’ pigs at weaning, which had been born in a different farrowing house. The resulting group of 230 pigs was divided between seven pens of 32–34 pigs (groups wn1–wn7; Table 3).

Sampling of the cohort next took place when the pigs were 18–19-week-old growers (visit 4, August). Upon leaving the weaner building six weeks earlier, tagged pigs from the original cohort were mixed into two groups, which differed in size and housing situation. Group gr1 comprised 101 pigs housed in a single large pen bedded on deep straw, and group gr2 comprised 43 pigs housed in a slatted-floor pen in a different building. The majority of untagged pigs that were sampled with the cohort during visit 2 were moved elsewhere on leaving the weaner building and not sampled again.

Final sampling took place one week prior to most cohort pigs being sent to slaughter (visit 5, October). However, group gr2 had already been sent to slaughter by this time and were not sampled again. Samples were collected from just one group of cohort pigs (gr1), which comprised 94 pigs housed in a single large pen bedded on deep straw. In contrast to the farm’s usual practice of moving pigs to smaller pens for the latter stages of fattening (see Section 2.1 and Table S1), these cohort pigs had been housed in a large stable group since leaving the weaner building ten weeks earlier.
