# Supplementary material for: Study of Animal Mixing and the Dynamics of Hepatitis E Virus Infection on a Farrow-to-Finish Pig Farm
Source: Animals (Basel). 2022 Jan 22;12(3):272. doi: 10.3390/ani12030272 (PMC8833537; doi:10.3390/ani12030272)
Supplement: Supplementary file 1 [file animals-12-00272-s001.zip › animals-1466765-supplementary.pdf]

Withenshaw et al. Study of animal mixing and the dynamics of Hepatitis E virus infection on a farrow-to-finish pig farm

# Supplementary Information

**Table S1:** Details of the usual pig herd management on the study farm

| Production stage | Age range (weeks) | Number of buildings | Number of pigs per pen | Floor type | All-in-all-out (AIAO) or continuous flow | Frequency of pen cleaning and disinfection |
|---|---|---|---|---|---|---|
| Piglets | 0 – 4 | 8 | 9 – 12 (+ sow in farrowing crate) | Semi-slatted | AIAO | Between batches |
| Weaners | 4 – 12 | 1 | 30 | Slatted | AIAO | Between batches |
| Growers | 12 – 20 | 5 | 30 – 180 | Most solid with shallow straw | Continuous flow | Twice per year |
| Fatteners | 20 – 25 | 4 | Most 10 – 20<br>3 pens 60 | Most solid with shallow straw | Continuous flow | Once per year |

**Figure S1:** A diagrammatic representation of the mixing of cohort pigs throughout the study and the corresponding timing of sampling visits. Details are also described in the text of Appendix C.

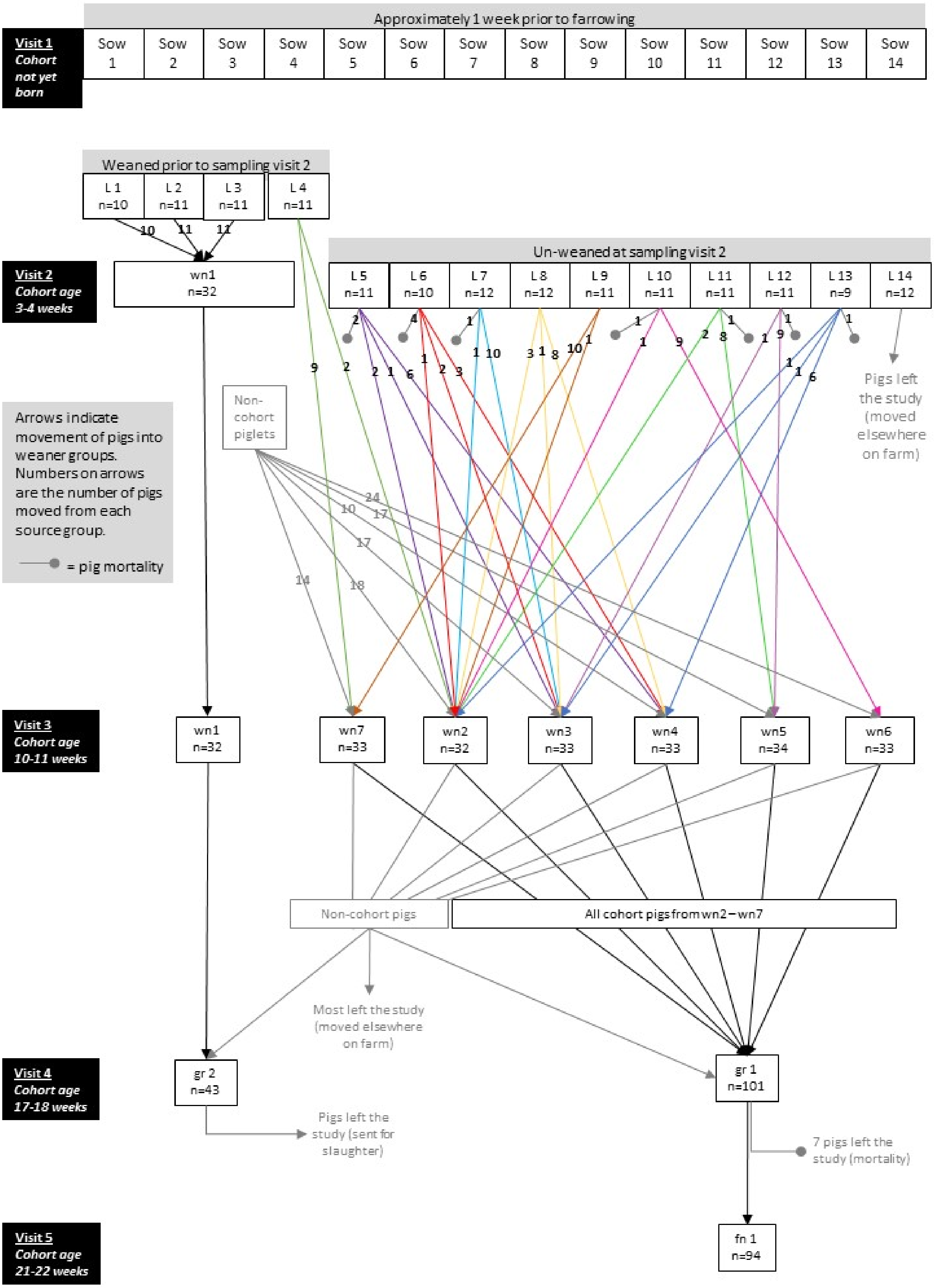

**Table S2:** Results of a multivariable logistic regression model (GLM) investigating the effect of pig stage and sampling month on the presence of HEV RNA in faeces collected at a farrow-to-finish pig farm. Samples collected from the study cohort are not included in this model. The model was built using a backwards stepwise process. The initial model included an interaction between pig stage and sampling month, but this was dropped from the final model due to non-significance ($\chi^2$=5.93, df=3, $p$=0.12).

| Variable | Effect likelihood [A] | | | Parameter estimates | | | OR (95% C. I.) [B] |
|---|---|---|---|---|---|---|---|
| | $\chi^2$ | d.f. | *p* | β | S.E. | *p* | |
| ***Intercept*** | - | - | - | -1.22 | 0.26 | <0.001 | - |
| ***Pig stage*** | 193.0 | 1 | <0.001 | - | - | - | - |
| Ref: Fatteners | | | | | | | |
| Growers | | | | 3.07 | 0.27 | <0.001 | 21.6 (13.1 – 37.1) |
| ***Sampling month*** | 19.44 | 3 | <0.001 | - | - | - | - |
| Ref: May | | | | | | | |
| July | | | | -0.81 | 0.35 | 0.02 | 0.45 (0.22 – 0.89) |
| August | | | | 0.49 | 0.34 | 0.15 | 1.64 (0.84 – 3.24) |
| October | | | | 0.53 | 0.32 | 0.10 | 1.70 (0.91 – 3.23) |

[A] Likelihood ratio $\chi^2$ test statistic, degrees of freedom (d.f.) and probability (*p*) of each fixed effect compared to a model containing the intercept and other fixed effect only.
[B] Odds ratios (and 95% confidence intervals) and *p*-values for the levels of each variable.

**Table S3:** Results of a two-way ANOVA investigating the effects of pig stage and sampling month on the viral load of HEV (copy number per gram) in the HEV-positive faeces of growers and fatteners on a farrow-to-finish pig farm. Samples from the study cohort are not included in this analysis. The dependent variable is a $\log_{10}$ transformation of HEV copy number per gram of faeces.

| Variable | d.f. | Sum sq. | Mean sq. | F | *p* |
|---|---|---|---|---|---|
| Pig stage | 1 | 87.22 | 87.22 | 97.97 | <0.001 |
| Sampling month | 3 | 9.94 | 3.31 | 3.72 | 0.01 |
| Pig stage*Sampling month | 3 | 2.98 | 0.99 | 1.12 | 0.34 |
| Residuals | 242 | 215.45 | 0.89 | - | - |

**Table S4:** Results of a post-hoc Tukey test based on the output of the ANOVA described in Table S2 to identify the sampling months that differed significantly in terms of the mean viral load of HEV in HEV-positive faeces collected from grower and fattener pigs on the study farm.

| Comparison | Difference [A] | Lower 95% CI [B] | Upper 95% CI [C] | *p (adj)* [D] |
|---|---|---|---|---|
| Growers - Fatteners | 1.33 | 1.06 | 1.59 | <0.001 |
| July – May | 0.15 | -0.33 | 0.63 | 0.85 |
| August – May | 0.52 | 0.08 | 0.95 | 0.01 |
| October – May | 0.36 | -0.06 | 0.79 | 0.11 |
| August – July | 0.37 | -0.10 | 0.84 | 0.17 |
| October – July | 0.22 | -0.24 | 0.67 | 0.61 |
| October – August | -0.15 | -0.56 | 0.25 | 0.76 |

[A] Difference between observed means
[B] Lower 95% confidence interval of the difference between means
[C] Upper 95% confidence interval of the difference between means
[D] *p*-value (adjusted for multiple comparisons) of the observed difference; significance level of $p \leq 0.05$ is assumed

**Table S5:** Results of a logistic regression model (GLM) investigating the effect of age on the presence of HEV RNA in faeces collected from a cohort of pigs at a farrow-to-finish pig farm. This model includes age as a fixed effect with two levels.

| Variable | Effect likelihood [A] | | | Parameter estimates | | | OR (95% C. I.) [B] |
|---|---|---|---|---|---|---|---|
| | β | S.E. | *p* | $\chi^2$ | d.f. | *p* | |
| ***Intercept*** | 0.31 | 0.19 | 0.09 | - | - | - | - |
| ***Visit number (age)*** | | | | 24.83 | 1 | <0.001 | |
| Ref: 11-12 & 17-18 weeks | | | | | | | |
| 21-22 weeks | -2.80 | 0.76 | <0.001 | | | | 0.06 (0.01 – 0.22) |

[A] Likelihood ratio $\chi^2$ test statistic, degrees of freedom (d.f.) and probability (*p*) of each fixed effect compared to a model containing the intercept and other fixed effect only.
[B] Odds ratios (and 95% confidence intervals) and *p*-values for the levels of each variable.

**Table S6:** Results of a one-way ANOVA investigating the effect of pig age on the viral load of HEV (copy number per gram) in the HEV-positive faeces of a cohort of pigs on a farrow-to-finish pig farm. The cohort was sampled at four time points (3-4 weeks, 11-12 weeks, 17-18 weeks and 21-22 weeks of age). No samples were positive for HEV at the first time point, therefore data from the first time point are not included in this analysis. The dependent variable is a $\log_{10}$ transformation of HEV copy number per gram of faeces.

| Variable | d.f. | Sum sq. | Mean sq. | F | *p* |
|---|---|---|---|---|---|
| Cohort age | 2 | 19.61 | 9.81 | 8.50 | <0.001 |
| Residuals | 70 | 80.87 | 1.15 | - | - |

**Table S7:** Results of a post-hoc Tukey test based on the output of the ANOVA described in Table A7 to identify the cohort age points that differed significantly in the mean viral load of HEV-positive faeces.

| Comparison (cohort age) | Difference [A] | Lower 95% CI [B] | Upper 95% CI [C] | *p (adj)* [D] |
|---|---|---|---|---|
| 17-18wks – 11-12wks | 0.61 | -0.09 | 1.31 | 0.10 |
| 21-22wks – 11-12wks | -2.28 | -4.20 | -0.36 | 0.02* |
| 21-22wks – 17-18wks | -2.89 | -4.74 | -1.04 | 0.001* |

[A] Difference between observed means
[B] Lower 95% confidence interval of the difference between means
[C] Upper 95% confidence interval of the difference between means
[D] *p*-value (adjusted for multiple comparisons) of the observed difference; significance level of $p \leq 0.05$ is assumed

**Table S8:** Results of three different univariable mixed-effects logistic regression models investigating risk factors associated with the occurrence of HEV in faecal samples collected from seven groups of 17-18 week old weaners sampled as part of a study cohort on a farrow-to-finish pig farm. All models included Group ID as a random effect.

| Fixed effect | Model *p* [A] | $\chi^2$ [A] | Random effect σ [B] | Fixed effect levels | ORs (95% C. I.) [C] | *p* [C] |
|---|---|---|---|---|---|---|
| Number of tagged litters (continuous variable) | 0.40 | 0.71 | 56.35 | Continuous | 3.02 (0.26 – 35.1) | 0.38 |
| Number of tagged litters (categorical variable) | 0.73 | 0.12 | 103.3 | *Ref:* 1-5 | - | - |
| | | | | >5 | 6.99 ($9.7\times10^{-5}$ – $5.1\times10^{5}$) | 0.73 |
| Proportion of tagged pigs [D] | 0.46 | 0.54 | 77.3 | Continuous | $2.19\times10^{5}$ ($6.5\times10^{-10}$ – $7.3\times10^{19}$) | 0.47 |

[A] Likelihood ratio $\chi^2$ test statistic and probability for each model compared to a null model containing just the intercept and the random effect
[B] Variance of the random effect for each model
[C] Odds ratios (and 95% confidence intervals) and *p*-values for each variable
[D] Proportion of tagged pigs is the number of pigs from the original study cohort (with ear tags) divided by the total number of pigs in the group, which may have also included pigs that were not part of the original study cohort (untagged)
